# The Role of Psychological Inflexibility in Adolescents' Loneliness: School Friendship Closeness as a Mediator

**DOI:** 10.1111/sjop.70053

**Published:** 2025-11-28

**Authors:** Annina Jormanainen, Kaisa Kalttila, Tetta Hämäläinen, Päivi Lappalainen, Mari Tunkkari, Noona Kiuru

**Affiliations:** ^1^ Department of Psychology University of Jyvaskyla Jyväskylä Finland

**Keywords:** educational transition, friendship closeness, loneliness, psychological inflexibility

## Abstract

This study aimed to expand knowledge on the roles of psychological inflexibility and school friendship closeness in adolescents' loneliness during the transition to upper secondary education. The participants were 885 Finnish adolescents (mean age = 15.74, SD = 0.37, 56% girls). Loneliness was measured twice: in Grade 9 and at the beginning of upper secondary school in 10th grade. Psychological inflexibility and the closeness of friendships in school were measured in Grade 9. Results showed that a high level of psychological inflexibility in Grade 9 was associated with higher loneliness at the beginning of upper secondary education and increased loneliness during the educational transition. Furthermore, girls' (but not boys') friendship closeness in school partly mediated the association between psychological inflexibility and loneliness. The results suggested that psychological inflexibility is a social risk factor for loneliness among adolescents transitioning to upper secondary school. Enhancing psychological flexibility skills could thus make an important contribution to the promotion of adolescent peer relationships and prevention of loneliness.

**Summary**

- We examined the roles of psychological inflexibility and school friendship closeness in adolescents' loneliness during the transition to upper secondary education.
- The results revealed that psychological inflexibility is a risk factor for increased loneliness during the transition.
- Furthermore, girls' (but not boys') friendship closeness in school partly mediated the association between psychological inflexibility and loneliness.

## 1 Introduction

In most educational systems, the transition from basic education to upper secondary education requires independence, making new social contacts, and adapting to a new learning environment. During these transitions, relationships with friends are changing due to the change of school, but at the same time, the importance of friends is increasing due to the need for autonomy and independence from one's family (Laursen and Hartl 2013; Steinberg 2016). Close friends are particularly important during adolescence because they provide emotional support, acceptance, and foster positive identity development, and may help to adapt to a new school environment (Benner et al. 2017; Eryılmaz et al. 2024; Steinberg 2016). However, about 8% of students in secondary education report not having any close friends (THL 2023). According to Erikson's (1993) psychosocial theory of development, the experience of loneliness can be particularly detrimental in adolescence because of social mirroring and the need for peer approval (Bishop and Keth 2013; Erikson 1993). One important factor possibly associated with friendships and loneliness is psychological inflexibility, which refers to an individual's inability to cope with, accept, and adjust to difficult situations (Hayes et al. 2006; Kashdan and Rottenberg 2010). The aim of this study was to increase understanding of the relationships between psychological inflexibility, loneliness, and friendship closeness during the transition from basic education to upper secondary education.

### 1.1 Loneliness and Friendships During Adolescence

*Loneliness* is defined as an unpleasant emotional response arising from the subjective feeling that one's needs for social relationships are not being met (Hawkley and Cacioppo 2010). According to the evolutionary theory of loneliness, the function of loneliness is to encourage individuals to engage in social interaction, which has been vital for survival (Cacioppo and Cacioppo 2018). Identifying loneliness with objective measures may be challenging because the feelings of loneliness depend on personal wishes for social relationships (Laursen and Hartl 2013; Lyyra et al. 2022). Therefore, even those adolescents who do see their friends can experience loneliness if their needs for friendships are not met (Nicolaisen and Thorsen 2017). Loneliness has been compared to physical pain, and it shares similar neural bases (Laursen and Hartl 2013).

Loneliness can be particularly detrimental for the development of adolescents as they attempt to find their social position and discover their social roles (Kiuru et al. 2025; Laursen and Hartl 2013; Steinberg 2016). Acceptance from the community and especially from friends is crucial for adolescent development because the sense of belonging to a group helps form a positive sense of self (Erikson 1993). Loneliness can hinder identity exploration, which includes social comparisons and feedback from peers (Eryılmaz et al. 2024; Kaniušonytė et al. 2019). Knowledge of the antecedents of adolescent‐perceived loneliness is vital for multiple reasons. It is known that adolescents have a heightened need to be close to and receive acceptance from friends (Laursen and Hartl 2013; Steinberg 2016). According to Secor et al. (2017), adolescents perceive that their friendships promote psychological well‐being during challenging life situations even more than their family relationships do. Young people also spend more time with their friends during adolescence than they do later in life (Nicolaisen and Thorsen 2017).

Unfortunately, loneliness among adolescents has increased in recent decades: In 2006 the prevalence was 11% and by 2018 it had risen to 15% (Lyyra et al. 2022). Moreover, loneliness has been associated with more frequent subjective health complaints (Lyyra et al. 2022) as well as lower physical and mental health in the long term (Hawkley and Cacioppo 2010). Loneliness during adolescence constitutes a significant risk factor for fulfillment of the need to belong as well as for normative development. Consequently, it is vital to study potential trajectories and risk factors for loneliness among adolescents to prevent ill‐being and social exclusion in adolescence and adulthood.

### 1.2 The Role of Psychological Inflexibility in Loneliness

*Psychological inflexibility* is a transdiagnostic process that involves rigid ways of reacting to one's negative thoughts, feelings, and experiences (Hayes et al. 2006; Levin et al. 2014). It has been found to be an important transdiagnostic process for poor mental health and a range of psychological disorders (Levin et al. 2014). Psychological inflexibility includes experiential avoidance (i.e., attempting to control or avoid unwanted internal experiences), poor self‐awareness (i.e., difficulties in separating oneself from emotions which may lead to impulse‐based actions), a rigid attitude toward one's own internal experiences, and value conflicts (Hayes et al. 2006). Psychological inflexibility can therefore be seen as difficulty in adapting to different changes. The opposite of psychological inflexibility is psychological flexibility, the primary target and process of change in acceptance and commitment therapy (ACT). Psychological flexibility can be understood as the capacity to be in contact with the present moment, fully aware and accepting of one's thoughts, emotions, and sensations, and to live according to their values (Hayes et al. 2006).

Some previous evidence for the connection between psychological inflexibility and loneliness has been presented by cross‐sectional single‐measurement surveys among adults (Bonilla‐Sierra et al. 2021; Carreno et al. 2023; Castro et al. 2021; Ortega Jiménez et al. 2021; Shi et al. 2016; Smith et al. 2019). According to a study by Castro et al. (2021), processes of psychological inflexibility, such as experiential avoidance and getting stuck in one's thoughts, were connected to higher loneliness. The results of Sujathamalini et al. (2025) also showed among 18–24 young adults that higher levels of psychological flexibility were associated with a lower level of lonelines. However, no prior studies exploring the associations between psychological inflexibility and loneliness have been conducted with younger adolescents in a longitudinal setting.

It seems possible that psychological inflexibility as a phenomenon may be connected to the experience of loneliness during an educational transition. This idea is supported by relational frame theory (RFT), which is the theoretical basis for ACT (Barnes‐Holmes et al. 2002; Hayes 2016; Torneke 2010). According to RFT, behavior in a particular environment may be contextually transferred to a similar situation. That is, negative experiences in previous social situations resulting from psychological inflexibility may begin to define behavior in similar situations. For example, psychological inflexibility has been associated with social anxiety, as previous negative experiences generally lead individuals to fear failure in social situations (Figueiredo et al. 2023; Tan et al. 2023). Thus, a person may even avoid situations where they could meet new people and expand their social support network. In an educational transition, this can mean that pre‐existing psychologically inflexible patterns and experiences of loneliness make it harder for an adolescent to make new friends, even in a new school environment.

Previous studies on the association between psychological inflexibility and increased loneliness during educational transitions are scarce. As an exception, Wols et al. (2015) examined the association between emotional skills and changes in loneliness after the educational transition to high school. It was found that deficits in understanding and controlling emotions were related to increased loneliness 10 months after the transition. In addition, loneliness was found to increase difficulties in processing emotions (Wols et al. 2015). However, although difficulties in dealing with emotions are part of psychological inflexibility, the connection between the whole concept of psychological inflexibility and changes in loneliness has yet to be studied.

### 1.3 Perceived Closeness With School Friends as a Mediator

Given that school is an important developmental context in adolescence (Eccles and Roeser 2011; Steinberg 2016), it is also important to consider the *closeness of school friendships* (Eryılmaz et al. 2024; Schwartz‐Mette et al. 2020). It is an essential factor for the emotional support, such as empathy and trust, that an adolescent receives in a friendship (Almquist et al. 2014). In close friendships, an individual can share their thoughts and receive support in various challenging situations, including educational transitions. Individuals who have close relationships also have higher psychological well‐being (Almquist et al. 2014; Eryılmaz et al. 2024).

From an ACT perspective, psychological inflexibility might have a negative impact on the quality of close relationships (Harris and Hayes 2019). It can, for example, hinder acting in social situations (Harris and Hayes 2019), and manifest itself in negotiations, conflicts, and discussions (Harris 2019). For example, the difficulty of being present can hinder giving emotional support and therefore have a negative impact on the closeness of friendships. There are no previous studies on the associations between psychological inflexibility and the closeness of friendships, but a few studies have addressed aspects related to these concepts. Gerhart et al. (2014) conducted a study with young adults and discovered that psychological inflexibility was one of the principal factors in social conflicts between individuals. Moreover, Agır (2018) found that adolescents' emotion management skills were positively associated with friendship quality.

It is also possible that the connection between psychological inflexibility and loneliness is partially or fully mediated by the closeness of friendship. Previous studies have shown that psychological inflexibility is related to higher loneliness (Carreno et al. 2023; Castro et al. 2021; Ortega Jiménez et al. 2021; Shi et al. 2016; Smith et al. 2019). The connection between psychological flexibility, the counterpart of psychological inflexibility, has also been reported in intervention studies with adults, suggesting that fostering openness and acceptance, a component of psychological flexibility, toward feelings of loneliness, may decrease loneliness and encourage greater engagement with others (Lindsay et al. 2019). In addition, the association between the closeness of friendships and loneliness has a strong empirical basis (Agır 2018; Schwartz‐Mette et al. 2020; Solmi et al. 2020). A novel subject is to study if the associations between psychological inflexibility and the development of loneliness are mediated through friendship closeness in school.

### 1.4 Research Questions and Hypotheses

**FIGURE 1:**
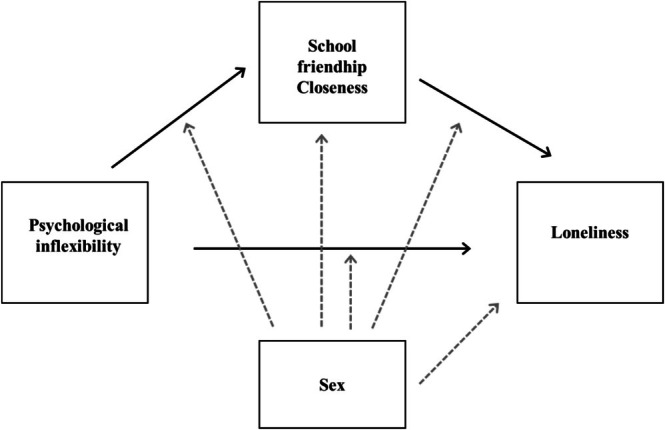
Schematic model of the aims of the present study.

This research aimed to expand the current knowledge on the roles of psychological inflexibility and school friendship closeness in adolescents' loneliness during the transition to upper secondary education. The schematic figure presented in Figure 1 illustrates the aims of the present study. More specific research questions and hypotheses were the following:

1. To what extent is psychological inflexibility in Grade 9 related to loneliness at the beginning of upper secondary education? Based on previous studies with adults (Ortega Jiménez et al. 2021; Shi et al. 2016; Smith et al. 2019) and ACT theory, it was hypothesized that psychological inflexibility in Grade 9 is associated with higher levels of loneliness in upper secondary education.
2. To what extent is psychological inflexibility related to the changes in loneliness during the transition to upper secondary education? It was expected that psychological inflexibility predicts increased loneliness during the transition (Wols et al. 2015).
3. To what extent is psychological inflexibility related to the school friendship closeness in Grade 9 and does school friendship closeness in Grade 9 mediate the association between psychological inflexibility and loneliness? Due to a lack of previous research on indirect associations, no specific hypotheses were set.

Previous studies have shown that psychological inflexibility tends to be higher among girls compared to boys (e.g., Liinamaa et al. 2022; Soares et al. 2023) and that girls tend to report loneliness more frequently than boys do (e.g., Lyyra et al. 2022), Adolescent biological sex was included as a control variable in subsequent analyses. Additionally, it was tested whether there are sex differences in the investigated associations (see also Figure 1).

## 2 Materials and Methods

### 2.1 Participants and Procedure

The present study is part of a broader longitudinal study following adolescents during the transition from basic education to upper secondary education. The participants were drawn from a large town and a middle‐sized town in Central Finland. These included semi‐rural areas. The participating schools were selected with the help of local school authorities from areas where all the students transfer to particular secondary schools instead of dispersing to different locations. In one of the towns all schools were recruited, whereas in the other town the selected schools covered about 75% of the targeted age group. Before the start of the longitudinal follow‐up study, all adolescents and their families were contacted and 74% of the families gave their informed consent to participate in the study. As the study progressed, families of students who transferred from other schools into the participating classes were also asked to provide consent for taking part in the study. In the ninth grade, there were 13 target schools. In addition, 32 students from the original sample had moved to other areas but chose to continue in the follow‐up.

This study focuses on two measurement points for which information on adolescents' loneliness was available: Grade 9 (Fall 2017, i.e., T1) and the first year in upper secondary education in Grade 10 (Spring 2019, i.e., T2). Data on psychological flexibility and school friendship closeness was collected at T1. The data were collected during normal school lessons. Written consent from the adolescents and their parents was required. The procedures of the study follow the principles of the Helsinki Declaration on research with human subjects and the study has been approved by the ethics committee of the Ethics committee of the University of Jyväskylä.

In the autumn of Grade 9 (T1), 885 adolescents participated in the study (*M* = 15.74, SD = 0.37; 56% females). Out of these 885 adolescents 679 (77%) participated in the study again in the first spring of upper secondary education (T2). At T2, 70% of the adolescents studied in an academic track, and 30% in a vocational track. A total of 77% of adolescents lived either in a nuclear family or stepfamily. Of the adolescents, 96% reported Finnish as their mother tongue. Information about the adolescents' mother tongue, family structure, and their parents' education is presented in Table 1.

**TABLE 1:** Demographic information of the sample.

		*n*	%
Mother tongue (*n* = 883)	Finnish	844	95.6
	Other	24	2.7
Living with (*n* = 869)	Mother and father	599	68.9
	Mother/father	93	10.7
	Alternatively with mother and father	92	10.6
	Mother/father and their new partner	70	8.0
	Foster care or approved home	4	0.5
Parents' education (*n* = 692)	University or university of applied sciences	313	45.3
	College of Professional Education	151	21.8
	Vocational education	209	30.2
	No vocational education	19	2.8

### 2.2 Measures

#### 2.2.1 Loneliness (T1 & T2)

Loneliness was measured with a single question on global loneliness, “Do you ever feel lonely?” on a four‐point scale: 1 = *yes, very often*, 2 = *yes, quite often*, 3 = *yes, sometimes* and 4 = *no*. The scale has been found to be reliable and valid by several studies (e.g., Lyyra et al. 2022; Mund et al. 2023; Pinquart and Sorensen 2001).

#### 2.2.2 Psychological Inflexibility (T1)

Psychological inflexibility was measured with the Acceptance and Fusion Questionnaire for Youth, AFQ‐Y8 (Greco et al. 2008; see also Lappalainen et al. 2021; Liinamaa et al. 2022; Livheim et al. 2016). The scale includes eight items (e.g., “I am afraid of my feelings”) that respondents rate on a five‐point scale (0 = *not at all true*; 4 = *totally true*). A sum score was calculated across the eight items on a scale of 0 to 32 (*M* = 8.28, SD = 6.57) and the Cronbach's alpha was 0.88.

#### 2.2.3 Closeness With School Friends (T1)

The closeness of the school friendship was measured using the seven‐question Friendship Qualities Scale (Bukowski et al. 1994; see also Kiuru et al. 2020). Adolescents were asked to think of their closest friend in their class or a parallel class and to rate their own experience of the closeness of the friendship (e.g., “I feel happy when I spend time with friends”) using a five‐point scale (1 = *not at all true*; 5 = *really true*). A mean score was calculated (*M* = 4.21, SD = 0.67) and its Cronbach's alpha was 0.87.

### 2.3 Attrition Analyses

Table 2 shows the differences between the adolescents who responded to the questionnaires in T1 and T2 and the ones who dropped out. The results of cross‐tabulation showed that compared to girls, boys were more likely to drop out of the study between T1 and T2 (*p* = 0.012, *χ* ^2^ = 6.287, df = 1). Of the participants, 80% of girls and 73% of boys responded to both surveys. The results also revealed that those who dropped out were slightly more psychologically inflexible. The effect sizes of these differences were nevertheless small (Cohen 1988). In terms of school friendship closeness and loneliness, those who dropped out were not significantly different from those who continued in the study.

**TABLE 2:** Differences between adolescents who answered at both measurement points and the ones who answered only at the first measurement point.

	Response at both measurement points	*N*	*M*	SD	*p*	Cohen *d*	*t*	df
Psychological inflexibility (T1)	No	203	9.56	7.616	< 0.001	0.254	2.839	286.702
	Yes	674	7.90	6.181				
School friendship closeness (T1)	No	199	4.18	0.692	0.374	−0.061	−0.760	856
	Yes	659	4.22	0.670				
Loneliness (T1)	No	199	1.7	0.778	0.936	0.058	0.715	862
	Yes	665	1.66	0.827				

*Note:* Cohen *d* = effect size: Effect size of 0.20 was considered small, 0.50 moderate, and above 0.80 large (Cohen 1988). *t* = test statistic, df = degrees of freedom, T1 = Grade 9, fall, T2 = spring of the first year of upper secondary education.

Abbreviations: *M*, mean; *p*, *p* value of the difference between groups; SD, standard deviation.

### 2.4 Statistical Analyses

Descriptive statistics were explored first. Regarding Cohen's *d*, the effect size of 0.20 was considered small, 0.50 moderate, and above 0.80 large (Cohen 1988). In turn, regarding the effect size of the correlations, an *r*‐value around 0.10 was considered a small effect size, around 0.30 a moderate effect size and above 0.50 a large effect size (Cohen 1988).

Next, research questions were answered using path analysis of structural equation modeling including adolescent gender as a control variable for all the analyses. The statistical analyses were performed using the Mplus statistical package (Version 8.9; Muthén and Muthén 1998–2023). The parameters of the models were estimated using full‐information maximum likelihood (FIML) with non‐normality robust standard errors (maximum likelihood estimator, MLR; Muthén and Muthén 1998–2023).

First, loneliness at the beginning of upper secondary education (T2) was predicted by adolescent gender and psychological inflexibility in Grade 9 (T1). Second, a change score of loneliness from Grade 9 to the first year of upper secondary education (T2–T1) was predicted by adolescent gender, an initial level of loneliness in Grade 9, and psychological inflexibility in Grade 9 (T1). Finally, mediation analysis was conducted to examine direct and indirect associations between psychological inflexibility and subsequent loneliness through friendship quality. A bootstrapping procedure with 95% confidence intervals (CI) was used to estimate the indirect associations (MacKinnon et al. 2004; Preacher and Hayes 2008). In the mediation analysis, it is assumed that variable X is associated with variable Y through variable M either partly or fully. The mediation analyses were carried out separately for girls and boys due to the gender differences in the associations of interest.

## 3 Results

### 3.1 Descriptive Statistics

Table 3 provides descriptive information on adolescents' loneliness. More than half of adolescents experienced loneliness at least sometimes in Grade 9 and upper secondary education. An independent samples *t* test showed that there was a statistically significant difference between girls and boys in their loneliness in both Grade 9 (*t*(860.024) = 6.846, *p* < 0.001, *d* = 0.48) and in the first year of upper secondary education (*t*(671) = 4.883, *p* < 0.001, *d* = 0.35). Girls experienced more loneliness than boys did at both measurement points (Table 3). The effect sizes of gender differences in loneliness ranged from small to moderate.

**TABLE 3:** Descriptive statistics on experiences of loneliness.

	All	%	Girls	%	Boys	%
*Loneliness (T1)*						
Never	421	48.7	194	39.7	227	60.5
Sometimes	339	39.2	215	44.0	124	33.1
Quite often	67	7.8	51	10.4	16	4.3
Very often	35	4.1	28	5.7	7	1.9
*M* (SD)	1.67 (0.79)		1.82 (0.82)		1.47 (0.64)	
*Loneliness (T2)*						
Never	312	46.4	144	36.4	168	60.6
Sometimes	264	39.2	186	47.0	78	28.2
Quite often	66	9.8	46	11.6	20	7.2
Very often	31	4.6	20	5.1	11	4.0
*M* (SD)	1.73 (0.82)		1.85 (0.81)		1.55 (0.80)	

*Note:* T1 = Grade 9, fall, T2 = spring of the first year of upper secondary education.

Table 4 shows correlations between the observed variables for the whole sample and separately for girls and boys. Psychological inflexibility correlated significantly with loneliness at both time points. Effect sizes of the correlations between psychological inflexibility and loneliness ranged from moderate to large. Correlations for closeness of friendship in school were different for girls and boys. For girls, school friendship closeness correlated significantly with other variables, albeit with relatively small effect sizes, while for boys the correlations were not significant.

**TABLE 4:** Correlations between variables.

	Psychological inflexibility			Loneliness (T1)			Loneliness (T2)		
	The whole sample	Girls	Boys	The whole sample	Girls	Boys	The whole sample	Girls	Boys
Loneliness (T1)	0.492***	0.502***	0.407***	1	1	1			
Loneliness (T2)	0.362***	0.397***	0.225***	0.479***	0.513***	0.356***	1	1	1
School friendship closeness (T1)	0.070*	−0.116*	0.046	0.016	−0.231***	0.006	0.008	−0.169***	−0.042

*Note:* T1 = Grade 9, fall, T2 = spring of the first year of upper secondary education. ***p < 0.001 and *p < 0.05. The whole sample *N* = 654–858, girls *N* = 386–484, boys *N* = 268–374. Correlation around 0.10 is considered a small effect size, around 0.30 a moderate effect size and above 0.50 a large effect size (Cohen 1988).

### 3.2 Psychological Inflexibility and Loneliness During the Educational Transition

Our first aim was to examine whether psychological inflexibility in Grade 9 is related to subsequent loneliness in the first year of upper secondary education. The path modeling results are shown in Table 5. The results showed that both gender and psychological inflexibility were significantly related to loneliness at the beginning of upper secondary education. Girls experienced loneliness more often than boys did. Furthermore, after accounting for the effect of gender, a higher level of psychological inflexibility in Grade 9 was associated with higher levels of subsequent loneliness in upper secondary education. Altogether, gender and psychological inflexibility explained 16% of the experiences of loneliness in the first year of upper secondary education.

**TABLE 5:** Results of path modeling of the associations between psychological inflexibility, gender, and loneliness.

Independent variables	Standardized *β*	SE	*p*
	Dependent variable: Loneliness (T2)		
Gender	−0.109	0.038	0.004
Psychological inflexibility (T1)	0.356	0.042	< 0.001
	Dependent variable: Change score of loneliness (T2‐T1)		
Gender	−0.058	0.036	0.111
Loneliness (T1)	−0.575	0.038	< 0.001
Psychological inflexibility (T1)	0.191	0.046	< 0.001

*Note:* 0 = girls, 1 = boys. T1 = Grade 9, fall; T2 = spring of the first year of upper secondary education.

### 3.3 Psychological Inflexibility and Changes in Loneliness During the Educational Transition

Our second aim was to examine the extent to which psychological inflexibility is related to the changes in loneliness during the transition from Grade 9 to the first year of upper secondary education. The path modeling results are shown in Table 5. The results showed first that, the lonelier an adolescent was in Grade 9, the more likely their loneliness increased from Grade 9 to the first year of upper secondary education. Gender did not significantly predict changes in loneliness after accounting for other factors in the model. In turn, over and above the effects of adolescent gender and an initial level of loneliness, psychological inflexibility predicted changes in loneliness during the educational transition. The more psychologically inflexible an adolescent was in Grade 9, the more likely they were to experience increased loneliness during the transition to upper secondary education. Overall, the model explained 25% of the change in loneliness from Grade 9 to the first year of upper secondary education.

### 3.4 Closeness With School Friends as a Mediator in the Associations Between Psychological Inflexibility and Loneliness

The first part of our third aim was to examine whether adolescents' psychological inflexibility is related to the closeness of their friendships in school. The results of path modeling, including gender, psychological inflexibility and the interaction term gender × psychological inflexibility as predictors of school friendship closeness, are presented in Table 6. The results showed that girls reported substantially higher school friendship closeness than boys. A higher level of psychological inflexibility was weakly related to a lower level of school friendship closeness. In addition, the interaction term gender × psychological inflexibility was statistically significant, suggesting that gender significantly moderated the association between psychological inflexibility and school friendship closeness. As earlier shown in correlations (Table 4), psychological inflexibility was related to friendship closeness only for girls (*β* = −0.12, *p* = 0.012) and not for boys (*β* = 0.05, *p* = 0.326). The more psychologically inflexible a girl was in Grade 9, the less close she experienced her relationship with her closest school friend. For boys, psychological inflexibility was not related to their friendship closeness. Overall, the model explained 26% of the variance of school friendship closeness.

**TABLE 6:** Results of path modeling of psychological inflexibility, gender, and their interaction term when predicting school friendship closeness.

Independent variables	Dependent variable: School friendship closeness (T1)		
	Standardized *β*	SE	*p*
Gender	−0.511	0.034	< 0.001
Psychological flexibility (T1)	−0.085	0.026	< 0.001
Gender × Psychological inflexibility (T1)	0.085	0.039	0.030

*Note:* 0 = girls, 1 = boys. T1 = Grade 9, fall.

**FIGURE 2:**
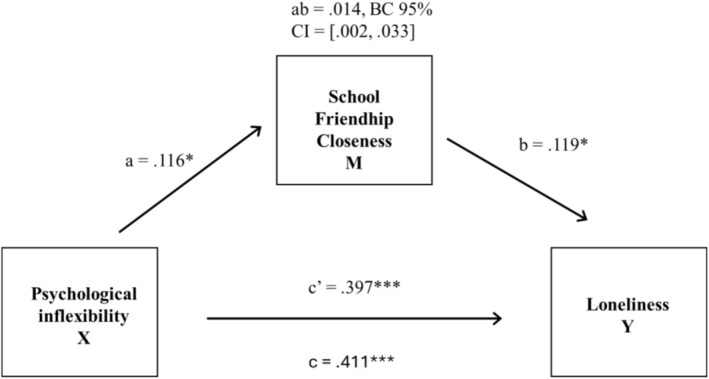
Mediation model of the association between girls' psychological inflexibility and loneliness, friendship closeness as a mediator. X = independent variable, M = mediator. Y = dependent variable, CI = confidence interval. Standardized estimates are presented. ***p < 0.001 and *p < 0.05.

Our final aim was to examine closeness with school friends as a mediator in the associations between psychological inflexibility and loneliness. The mediator analyses were carried out separately for girls and boys due to the gender differences in the associations of interest (Tables 4 and 6). Significant associations were found for girls (see Figure 2). The results for girls showed that psychological inflexibility was associated with loneliness through school friendship closeness (route *ab*). The association was significant both directly (route *c'*) and indirectly (route *c*, *c* = *ab + c*'). Girls' school friendship closeness was one of the factors that was related to the association between psychological inflexibility and loneliness. Psychological inflexibility was negatively associated with school friendship closeness (route *a*) as earlier noted. Friendship closeness and loneliness were negatively associated (route *b*); that is, the girls who assessed their school friendship as less close also experienced more loneliness. However, a statistically significant explanatory power remained for the direct association (route *c'*), meaning that friendship closeness partially mediated the link between psychological inflexibility and loneliness.

**FIGURE 3:**
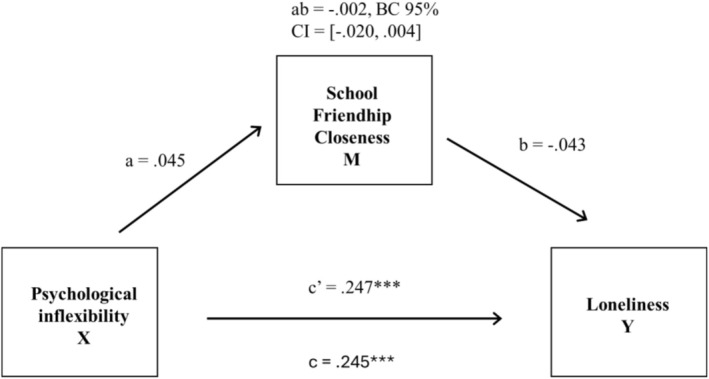
Mediation model of the association between boys' psychological inflexibility and loneliness, friendship closeness as a mediator. X = independent variable, M = mediator, Y = dependent variable, CI = confidence interval. Standardized estimates are presented. ***p < 0.001.

The mediation (route *ab*) was not significant for boys (see Figure 3). However, the direct association between psychological inflexibility and loneliness was significant (route *c'*). The results showed that psychological inflexibility and loneliness in upper secondary education were associated for boys too, but school friendship closeness did not mediate the association of these factors.

## 4 Discussion

This study explored the association between psychological inflexibility and loneliness during the transition from Grade 9 of basic education to upper secondary education. In addition, school friendship closeness was examined as a mediator between these two concepts. This research topic is important since much is known about the negative longitudinal effects of loneliness, but research on the link between psychological inflexibility and loneliness is scarce. The analyses revealed significant differences between genders, which became important particularly when examining the closeness of school friendships.

### 4.1 The Role of Psychological Inflexibility in Loneliness During the Educational Transition

The results were in line with our hypothesis and previous literature (Carreno et al. 2023; Ortega Jiménez et al. 2021; Shi et al. 2016; Smith et al. 2019) that psychological inflexibility in Grade 9 was associated with adolescents' subsequent experiences of loneliness at the beginning of upper secondary education. In addition, consistent with the second hypothesis (Wols et al. 2015), the more psychologically inflexible an adolescent was in Grade 9, the more likely they were to experience an increase in loneliness during the transition to upper secondary education. The effect sizes for the associations between psychological inflexibility and loneliness ranged from moderate to large. These results are new, as the association between psychological inflexibility and loneliness has not been previously studied among adolescents transitioning to upper secondary education.

These results align with the ACT perspective, suggesting that psychological inflexibility affects individuals' suffering and interpersonal behavior (Hayes et al. 2006). The results may therefore be influenced by different processes of psychological inflexibility (see Castro et al. 2021). For example, one aspect of psychological inflexibility, such as being stuck in one's thoughts, may also be a factor contributing to loneliness during educational transitions (Castro et al. 2021). Adapting to a new environment is often stressful and can trigger unpleasant feelings such as anxiety. Psychological inflexibility may increase anxiety and the tendency to get attached to one's thoughts in such situations. This can weaken an adolescent's ability to be fully present in a new social situation, which can make it more difficult to connect with other people. Castro et al. (2021) also found that experiential avoidance was associated with loneliness. Avoiding difficult thoughts, emotions and situations may also influence experiences of loneliness in a new school. An adolescent may withdraw and avoid meeting new people because they want to avoid the unpleasant feelings the situation causes. Moreover, suppressing emotions undermines the experience of positive emotions as well (Kashdan et al. 2006; Philippot and Feldman 2004), which may, in turn, affect the experience of loneliness or how much pleasure one gets from relationships. Getting to know other people may also be complicated by poor self‐perception, which is related to psychological inflexibility. Adolescents' difficulty in recognizing their feelings may be reflected in their relationships. On the other hand, an adolescent may also become stuck in the feeling of loneliness without being able to identify the negative feelings arising from the loneliness or how to respond to them.

Moreover, as RFT (e.g., Hayes 2016) suggests, previous negative experiences in social situations may influence an adolescent's behavior in similar situations. For example, psychological inflexibility and related problems in social relationships may increase avoidance behavior and thus decrease the courage to meet new people in a new school. On the other hand, an adolescent may also be more likely to give up on meeting new people if they fail to make friends immediately. Psychological inflexibility is also associated with the belief in self‐immutability (Harris and Hayes 2019). Loneliness may be maintained by the fact that the loneliness experienced in lower secondary school has become a defining role for an adolescent in the community. It is easy to continue this role in a new environment, and it can be difficult to break the negative cycle if that adolescent does not believe the feelings of loneliness can be reduced. The constant experience of loneliness can also interfere with identity formation, and thus potentially harm the adolescent's development (Kaniušonytė et al. 2019). Problems during an educational transition may also carry a risk of reoccurring in the future, such as when transitioning to work.

Psychological inflexibility may also affect the way others see the adolescent (Harris and Hayes 2019). For example, avoidance behavior may give others the impression that an adolescent does not want to interact socially with others. On the other hand, in social situations, being stuck in one's own thoughts and having difficulty being present may be seen by others as indifference or unwillingness to engage in conversation. In addition, poor emotional skills can also make it difficult to understand others' feelings and react to situations in a socially accepted way, which can make people feel offended.

### 4.2 Perceived Closeness With School Friends and Psychological Inflexibility

The third research question explored the association between psychological inflexibility and school friendship closeness. The results showed that the association between inflexibility and school friendship closeness was negative and statistically significant for girls but not for boys. One explanation for our findings could be drawn from girls' and boys' characteristic differences in friendships. It could be that psychological inflexibility has a stronger role in girls' school friendships because thoughts and worries are shared more often in girls' friendships. There tend to be more overthinking, corumination, and negative problem talk in girls' friendships (Schwartz‐Mette and Rose 2012). Girls appreciate closeness and discussions with their friends while boys prefer enjoyment, spending time, and doing activities together (Rudolph and Dodson 2022). According to Almquist et al. (2014), sharing personal issues with a friend correlated to friendship quality more strongly for women than it did for men. The possible negative effects of inflexibility may be more pronounced in girls' communication, whereas for boys the effects of inflexibility may remain lower due to a lower level of self‐disclosure.

The final research question examined whether friendship closeness could mediate the association between psychological inflexibility and loneliness. The results showed that for girls, weaker closeness of school friendship partly explained the connection between psychological inflexibility and loneliness. Girls' psychological inflexibility was related to weaker school friendship closeness in Grade 9, which was related to higher loneliness at the beginning of upper secondary education. For boys, however, closeness of friendship did not mediate the association.

Gender differences in the results may be influenced by personal values, as girls in general perceive close relationships to be more important than boys do. According to Thomas and Daubman (2001), high school–aged girls evaluate their friendships as more rewarding but also as more stressful than boys do. Moreover, friendships have been found to influence women's subjective well‐being more strongly than they do that of men (Lu et al. 2021). Gender differences in the role of friendship closeness between psychological inflexibility and loneliness may be present because boys may be less affected by the lack of close relationships. Miljkovitch et al. (2021) found that 14‐year‐old boys relied on their parents more than their friends while girls relied more on their friends. Differences between girls and boys in peer relations (and their importance) may be one explanation why gender‐based differences were also found in the association between friendship closeness and inflexibility.

School context can also influence gender‐related differences. In this study, the focus was on friendships at school so it may also be that girls perceive their friendships at school to be more important than boys do. In contrast, the importance of friends in leisure time can be more pronounced for boys. For example, a sports team or gaming friends may have greater importance for boys (Rudolph and Dodson 2022). During the transition to upper secondary education, the role of friendships at school may not be as apparent for boys if the friendships formed through hobbies remain the same. However, close friendships may also continue in leisure time and help reduce feelings of loneliness in upper secondary education.

## 5 Limitations and Future Directions

Despite the longitudinal setting and from moderate to large effect sizes for the first two research questions, the study was a correlative one, meaning that no causal inferences can be drawn. Future studies could also assess whether gender differences discovered in this study are present in other age groups as well. It should also be noted that this study assessed gender based on biological sex. This means it would be important in the future to consider the experiences and possible differences between other gender identities as well. It is also important to note that this study focused only on the closeness of friendships in the school environment and that effect sizes of school friendship closeness (research question 3) were small. In the future, friendships outside of school and in other contexts such as social media could also be included to obtain a more comprehensive view of the closeness of adolescents' friendships. Our design also comprised only two time points with X (psychological inflexibility) and M (friendship closeness) variables measured at T1 and Y variable (loneliness) both at T1 and T2. This precluded us from investigating longer mediator chains and making conclusions about the causal mediating effects. Hence, further studies using more than two time points are needed to replicate the findings but also to investigate the longitudinal aspects in mediation (see also Caemmerer et al. 2024). In addition, further investigation of loneliness and the processes of inflexibility and flexibility (see also Castro et al. 2021) during educational transitions could possibly reveal if targeting specific flexibility processes could be beneficial in alleviating adolescent experiences of loneliness. Future studies could also address the topic in more detail in terms of adolescent autonomy development, identity development, and self‐development.

## 6 Conclusions

This study provided new insights into the association between psychological inflexibility and loneliness among adolescents transitioning to upper secondary education. The results revealed that psychological inflexibility is a risk factor for increased loneliness during the transition. It was also found that the connection between psychological inflexibility and loneliness is partially mediated through the closeness of school friendships in girls. The study reinforces the idea that psychological flexibility is an important factor in the functioning of interpersonal relationships.

It is important to train psychological flexibility at an early stage (for an example, see the Magis mobile game in Keinonen et al. 2023). In addition, various online interventions for adolescents have been developed to increase psychological flexibility, such as the Youth Compass (see, e.g., Lappalainen et al. 2021). Adding such inexpensive and easily accessible interventions to schools would allow children and adolescents to learn ways of dealing with future difficult situations, such as educational transitions. This study suggests that increasing the skills practiced in these interventions could also be important for preventing loneliness and improving the closeness of friendships, especially among girls.

## Author Contributions

A.J. and K.K. conceived the study, analyzed the data and drafted the manuscript. T.H., P.L. and M.T. participated in the interpretation of the data and helped to draft the manuscript. N.K. participated in the design, coordination, and interpretation of the data and helped to draft the manuscript. All authors read and approved the final manuscript.

## Ethics Statement

This study was conducted in compliance with APA ethical standards. The procedures were in accordance with the principles of the Helsinki Declaration on research with human subjects. The research plan of the project was approved by the Human Sciences Ethics Committee of the University of Jyväskylä.

## Consent

Informed consent to participate and publish the study results was obtained from all the participants of the study.

## Conflicts of Interest

The authors declare no conflicts of interest.

## Data Availability

The data that support the findings of this study are available on request from the corresponding author. The data are not publicly available due to privacy or ethical restrictions.
